# Quality of complementary and alternative medicine information for type 2 diabetes: a cross-sectional survey and quality assessment of websites

**DOI:** 10.1186/s12906-021-03390-3

**Published:** 2021-09-17

**Authors:** Jeremy Y. Ng, Manav Nayeni, Kevin Gilotra

**Affiliations:** Department of Health Research Methods, Evidence, and Impact, Faculty of Health Sciences, Michael G. DeGroote Centre for Learning and Discovery, McMaster University, Room 2112, 1280 Main Street West, Hamilton, Ontario L8S 4K1 Canada

**Keywords:** Complementary and alternative medicine, Consumer health information, Diabetes, DISCERN, Information assessment, Quality of information, Type 2 diabetes mellitus

## Abstract

**Background:**

The global prevalence of diabetes mellitus is projected to reach approximately 700 million by the year 2045, with roughly 90–95% of all diabetes cases being type 2 in nature. Patients with type 2 diabetes mellitus (T2DM) frequently seek information about complementary and alternative medicine (CAM) online. This study assessed the quality of publicly accessible websites providing consumer health information at the intersection of T2DM and CAM.

**Methods:**

An online search engine (Google) was searched to identify pertinent websites containing information specific to CAM for T2DM patients, and the relevant websites were then screened with an eligibility criteria. Consumer health information found on eligible websites were then assessed for quality using the DISCERN instrument, a 16-item standardized scoring system.

**Results:**

Across the 480 webpages identified, 94 unique webpages remained following deduplication, and 37 eligible webpages belonged to and were collapsed into 30 unique websites that were each assessed using the DISCERN instrument. The mean overall quality score (question 16) across all 30 assessed websites was 3.55 (SD = 0.86), and the mean summed DISCERN score was 52.40 (SD = 12.11). Eighty percent of websites presented a wide range of CAM treatment options with the associated benefits/risks of each treatment, but in 56.7% of the websites, the sources used to collect information were unreliable.

**Conclusion:**

This study identified, assessed, and presents findings on the quality of online CAM information for T2DM. Although there were several high scoring websites, there was variability across most of the individual DISCERN items in the assessed websites. This study highlights the importance of awareness among healthcare providers regarding the reliability of online information about CAM treatment and management options for T2DM. Healthcare providers should be aware of patients' information seeking behaviour, guide them in navigating through the content they encounter online, and provide them with resources containing trustworthy and reliable information.

## Background

According to the International Diabetes Federation, the global prevalence of diabetes mellitus was approximately 439 million in 2019 [1]. Type 2 diabetes mellitus (T2DM) roughly accounts for 90–95% of all cases [2]. The global prevalence of diabetes mellitus is projected to reach approximately 700 million by the year 2045 [1]. T2DM is a metabolic disorder characterized by insulin resistance, which refers to a diminished response to the hormone insulin in the body [3]. Insulin plays a crucial role in glucose homeostasis by reducing blood glucose levels [4]. Patients with T2DM typically display elevated blood glucose levels [5]. Treatment for T2DM typically includes lifestyle changes as well as the use of oral hypoglycaemics to promote glucose uptake [5, 6]. The chronic nature of T2DM can have adverse physiological effects on individuals over time including retinopathy, neuropathy and diabetic ulcers [3].

Patients diagnosed with chronic conditions, including T2DM, often seek complementary or alternative medicine (CAM) to manage their health [7]. As an example, it has been found that over 79% of Canadians had used CAM at some point in their life [8]. CAM refers to practices and therapies that are not extensively taught in Western medical schools nor typically utilized in conventional medicine; specifically, “complementary medicine” includes non-conventional practices used in *conjunction* with conventional care, while “alternative medicine” refers to non-conventional practices used in *replacement* of conventional ones [9–11]. Some examples of frequently used CAM therapies for T2DM include acupuncture, mind-body interventions, and dietary supplements [12, 13]. According to a 2018 nationwide survey within the United States, 57% of patients with T2DM have used CAM [13]. Oftentimes, patients consult the internet as an avenue for identifying potential CAM treatment options for their health conditions [14]. The internet is the primary source of CAM information for many patients, making it important to determine the quality of the information accessible to the general public [14]. To our knowledge, no prior research has identified nor assessed the quality of consumer health information presented online for CAM-specific treatment and/or management options for T2DM, thus this is the purpose of this study.

## Methods

### Search strategy and screening

A search strategy was developed to yield websites; Google was the sole websites searched, as it is the most commonly used search engine globally [15]. The search terms we selected were designed to reflect a “typical” patient’s information-seeking behaviour regarding CAM for T2DM. Four search terms were searched as follows: “alternative medicine for diabetes”, “complementary and alternative medicine for diabetes”, “complementary medicine for diabetes” and “integrative medicine for diabetes”. Searches were conducted using the Google Chrome browser in incognito mode, to ensure that searches were not affected by previous browser search histories. Through the search settings, the Google location was changed to include results from Australia (Google.com.au), Canada (Google.ca), the United Kingdom (Google.co.uk) and the United States (Google.com), to reflect a more internationally representative sample of websites.

### Eligibility criteria

Websites were screened for eligibility and were included if they contained CAM consumer health information pertaining to the treatment and/or management of T2DM. KG and another research assistant screened all results from the first two Google pages for each search term, and duplicate websites were removed. The websites also had to be freely available to the general public and written in the English language. Websites were excluded if they were peer-reviewed journals or articles, websites of major consumer retailers (eBooks, Google Books, etc.) as these are pay-to-view, were broken URLs, were from online newspapers that did not provide consumer health information or were exclusively video sites (i.e., YouTube). Different webpages captured from the same website were collapsed into a single item, as we applied the DISCERN instrument to information found across websites, and not individual webpages [16].

### Data extraction and website quality assessment

KG and the other research assistant extracted the following background information from eligible websites: the website name and URL, types of CAM and non-CAM therapies mentioned, whether the website appeared in more than one search and whether the website was certified by Health on the Net Foundation Code of Conduct (HONcode). We also classified each eligible website into one of five categories as follows: health portal, professional, news, government or other. The professional category encompassed websites developed by healthcare professionals/experts or health organizations. The health portal category included websites that presented information about a range of health topics. The government category included websites belonging to official government organizations and bodies. The news category consisted of websites from media sources such as newspapers, magazines or television stations and that were created for the distribution of current events. Lastly, the other category included any other websites that did not fall into any of the previously listed categories. We then assessed the quality of consumer health information available on each eligible website using the DISCERN instrument. The DISCERN instrument is comprised of 16 items divided into three sections, each of which aims to assess the quality of written consumer health information [16]. The first section, comprised of questions 1–8, assesses the reliability of the information presented. The second section, comprised of questions 9–15, assesses the quality of the information presented as treatment choices. Lastly, the third section is comprised of a singular question, question 16, and asks the user to provide an overall rating for the resource in its entirety. Each individual question was scored on a Likert scale from one to five, whereby one represented the lowest quality and five the highest quality [16].

To standardize the scoring process using the DISCERN instrument, JYN, KG and the other research assistant conducted a pilot test on three separate websites and used the DISCERN instrument to score the websites. Following the pilot test, all three assessors met to compare their scores and resolve any discrepancies to ensure the standardization of scores and that the DISCERN instrument was applied accurately according to the manual’s instructions. Following the standardization, KG and the other research assistant scored each eligible website using the instrument. Following scoring, JYN met with the two assessors and all discrepancies between the scores of the two assessors were resolved, without unduly modifying scores. MN averaged the two assessors’ scores for each question across all eligible websites, and a summed DISCERN score between values 15–75 was tabulated from questions 1–15. MN then calculated the average and standard deviation for each score, alongside an average score for all 16 items, and JYN reviewed all calculations. It is important to note that while the DISCERN instrument can be used to judge the reliability of a website as a source of information about treatment choices, it cannot be used to assess the scientific quality or accuracy of the evidence on which a publication is based, as this would require checking the information presented on each website against other sources [16].

## Results

### Search results

**Fig. 1:**
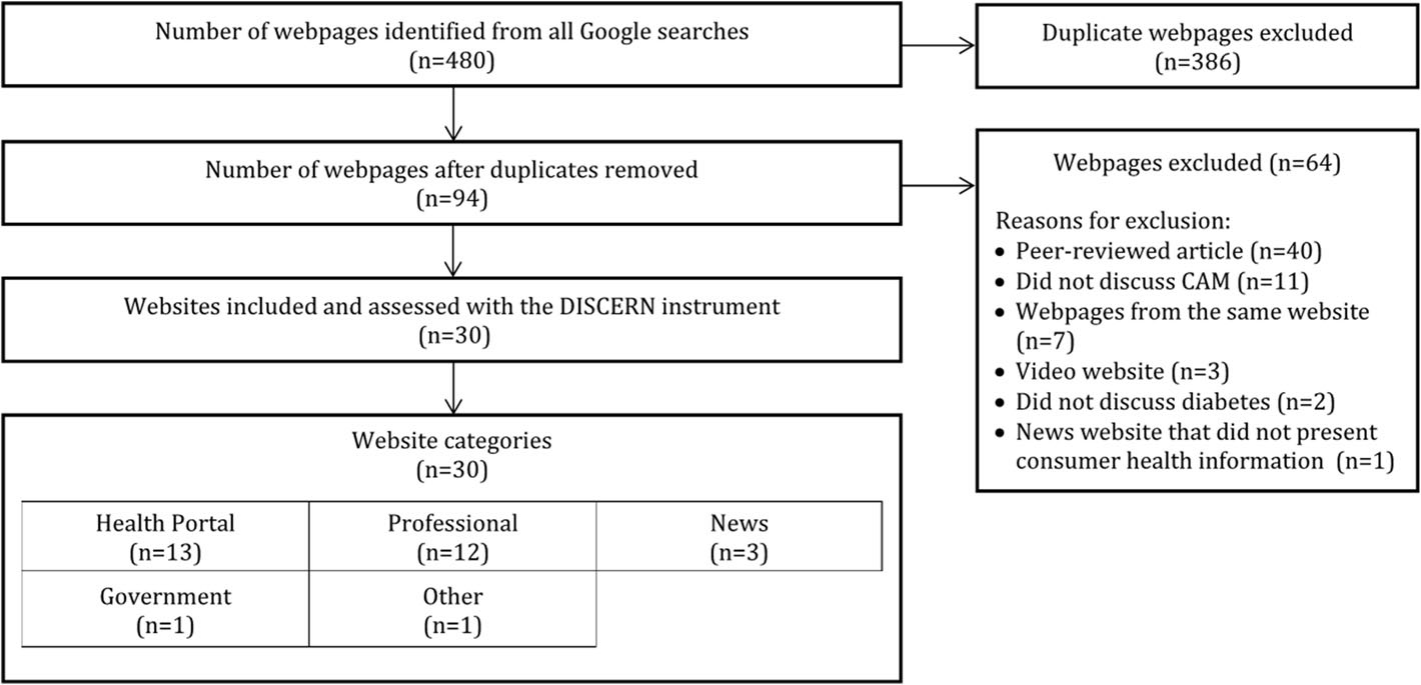
Web Information Search Strategy and Assessment Flowchart

Of the 480 webpages returned from the Google searches, 94 unique webpages remained following deduplication. Of these, 64 webpages were excluded for the following reasons: were peer-reviewed articles (*n* = 40), did not discuss any CAM therapies and/or recommendations (*n* = 11), were webpages from the same website (*n* = 7), were video websites (*n* = 3), did not discuss diabetes (*n* = 2), or was a news website that did not provide consumer health information (*n* = 1). The remaining 30 websites were then assessed using the DISCERN instrument. A flowchart of the search and screening process illustrated in Fig. 1.

### General characteristics of eligible websites

We categorized the 30 eligible websites as follows: health portals (*n* = 13), professional (*n* = 12), news (either online magazines/TV channels) (*n* = 3), government (*n* = 1), and other (*n* = 1). Commonly mentioned CAM therapies across websites included the following: acupuncture (*n* = 12), chromium (*n* = 11), yoga (*n* = 9), magnesium (*n* = 6) and aloe vera (*n* = 5). The most common non-CAM therapies mentioned across the websites included selective sodium-glucose transporter-2 inhibitors (*n* = 3), thiazolidinediones (*n* = 2), dipeptidyl peptidase-4 inhibitors (*n* = 2) and dietary monitoring (*n* = 2). A detailed account of the general characteristics of the eligible websites are shown in Table 1.

**Table 1:** General characteristics of eligible websites

Website Name	URL	Website Category	Types of CAM Discussed	Types of Non-CAM Therapies Discussed	Appeared in More than One Search?
Alt-Med	http://www.alt-med.org	Professional	Select supplement and herbs, stress reduction therapies, reasonable lifestyle changes	None	No
Association of Traditional Medicine and Acupuncture UK	https://www.atcm.co.uk	Professional	Acupuncture, huangqi, lugen, tianhaufen, huangbai, zexie, danzhuyet	None	No
Canadian College of Naturopathic Medicine	https://rsnc.ca	Professional	Chromium, homeopathy, traditional Chinese medicines, botanical medicine	None	No
Chelation	https://chelation.me	Other	Gluten free diet, coffee, oolong tea, yoga, tai chi, meditation, vitamin C, magnesium, chromium, biotin, vitamin D, alpha lipoic acid, bitter melon, fenugreek, *G. sylvestre*, cinnamon, Gui zhi, ginseng, berberine, *Gaertn sulyonarin*, *Shenyan kangfu*	Dietary monitoring, thiazolidinediones, dipeptidyl peptidase-4 inhibitors, selective sodium-glucose transporter inhibitors, insulin, chelation therapy	No
Conscious Lifestyle Magazine	https://www.consciouslifestylemag.com	Online magazine	Essential dietary oils, polyphenols, chromium, magnesium, R- alpha lipoic acid, botanical medicines, *G. sylvestre*, cinnamon, green tea leaf extract, curcumin extract	None	No
Diabetes Australia	https://www.diabetesaustralia.com.au	Government	Homeopathy, guanmu tong	None	No
Diabetes in Control	http://www.diabetesincontrol.com	Professional	Herbal therapies, chiropractic therapy, massage therapy, acupuncture, yoga, meditation	None	No
Diabetes Self Management	https://www.diabetesselfmanagement.com	Health Portal	Acupuncture, hypnosis, acupressure, qigong, mindfulness, ayurveda	None	No
Diabetes.co.uk	https://www.diabetes.co.uk	Health Portal	*A. vera*, bilberry extract, bitter melon, cinnamon, fenugreek, Ginger, okra allium, *B. forficata*, *Myrcia uniflora*, *C. indica*, *F. carica*, ginseng, *G. sylvestre*, *M. charantia*, *O. sanctum*, *O. streptacantha*, *Trigonella foeunum graecum,* berberine cinnamomum, tamala curry, *E. jambolana*, gingko, *P. amarus*, *P. marsupium*, *S. torvum*, *V. rosea*	None	Yes
Diabetic Nation	https://diabeticnation.com	Health Portal	Herbal therapy, aromatherapy, yoga, meditation, acupuncture, acupressure, massage therapy	None	No
Dr. Kirsten Smith, ND	https://www.drsmithnd.com	Professional	Botanical medicine, traditional Chinese medicines	None	No
Dr. Axe	https://draxe.com	Professional	Chromium picolinate, magnesium, healthy fats, low glycemic diet, cinnamon, fish oil, alpha lipoic acid, bitter melon, exercise, yoga	None	No
EndocrineWeb	https://www.endocrineweb.com	Professional	Low glycaemic diet, chromium, stress reduction, acupuncture, alpha lipoic acid, omega-3 fatty acids, polyphenols	None	No
Endocrinology Network	https://www.endocrinologynetwork.com/view/alternative-and-complementary-medicine-diabetes	Professional	Chromium, *G. sylvestre*, bitter melon, aloe vera, fenugreek, cinnamon, vanadium, omega-3, vitamin D, acupuncture, tai chi, qigong, yoga, laughter therapy	None	Yes
Everyday Health	https://www.everydayhealth.com	Health Portal	Exercise, yoga, stress management	Sleep apnea devices	Yes
Healthline	https://www.healthline.com	Health Portal	Exercise, low carb diet, stress management, yoga, mindfulness exercises, chromium, magnesium, apple cider vinegar, cinnamon, berberine, fenugreek	None	Yes
Institute for Natural Medicine	https://naturemed.org	Health Portal	Herbal substitutes	Insulin	No
Medical News Today	https://www.medicalnewstoday.com	Health Portal	*A. vera*, cinnamon, bitter melon, milk thistle, fenugreek, gymnema, ginger	None	No
NDTV Food	https://food.ndtv.com	Online TV station website	Fenugreek seeds, neem, turmeric powder, bitter gourd, gurmar	None	Yes
On Track Diabetes	https://www.ontrackdiabetes.com	Health Portal	Homeopathy, acupuncture, supplements, yoga, massage, organic food diet	None	No
Onyx Integrative Medicine and Aesthetics	https://onyxintegrative.com	Professional	Acupuncture, mind-body medicine, exercise, supplements	None	No
Practo	https://www.practo.com	Health Portal	Bitter gould, fenugreek, mango leaves, Indian gooseberry, moringa leaves	None	No
Stamford Health	https://www.stamfordhealth.org	Professional	Apple cider vinegar, barley, chromium, zinc, aloe vera, berberine, cinnamon, fenugreek, gymnema, nopal	None	No
The Health Site	https://www.thehealthsite.com	Health Portal	Tulsi leaves, flax seeds, bilberry leaves, cinnamon, green tea, drumstick leaves, psyllium husk, bitter gourd, neem, Indian blackberry	None	No
Today’s Dietitian	https://www.todaysdietitian.com	Online magazine	Gluten free diet, fish oils, yoga, qigong	None	No
Totally Health	https://www.totallyhealth.com	Health Portal	Acupuncture, Chinese herbal medicines, Ayurvedic medicines, vitamin D, aromatherapy, relaxation therapy, guided imagery, massage therapy, homeopathy, biofeedback	None	No
University of Minnesota	https://www.takingcharge.csh.umn.edu	Professional	Acupuncture, moxibustion, Chinese herbal medicines, diet therapy, qigong, tai chi, tui na, *P. ginseng*, *Momodica charantia*, *Lageneria siceraria*, *Psidium gnajava*, alpha lipoic acid, essential fatty acids, vitamin C, *ginkgo biloba*, mind-body practices, stress management	Insulin, exercise	No
University of Rochester Medical Centre	https://www.urmc.rochester.edu	Professional	Alpha lipoic acid, chromium, polyphenols, ginseng, acupuncture, exercise	Selective sodium-glucose transporter 2 inhibitors	No
Very Well Health	https://www.verywellhealth.com	Health Portal	Ginseng, chromium, magnesium, cinnamon, aloe vera, gymnema	Exercise, portion control, insulin, biguanides, thiazolidinediones, alpha-glucosidase inhibitors. Meglitinides, dipeptidyl peptidase-4 inhibitors, selective sodium-glucose transporter inhibitors, incretin mimetics, amylin analogue, bariatric surgery	No
WebMD	https://www.webmd.com	Health Portal	Acupuncture, vanadium, magnesium, ginseng, chromium, biofeedback, guided imagery, chitosan, *Garcinia cambogia*, pyruvate, germander, *M. charantia*, *S. androgynus*, aristolochic acid	Insulin	No

### DISCERN instrument ratings

The average summed DISCERN score across the eligible 30 websites was 52.40 (SD = 12.10). The average overall quality score for all the websites (question 16) was 3.55 (SD = 0.86). The three websites with the highest DISCERN score were as follows: WebMD (69.00), Diabetes Self Management (67.50) and Diabetes.co.uk (66.50). These three websites scored consistent 4s and 5s across all 16 DISCERN items. The three poorest scoring websites were as follows: Onyx Integrative Medicine and Aesthetics (32.50), Canadian College of Naturopathic Medicine (32.00) and NDTV Food (31.00). These websites consistently scored 2s and 3s across most of the DISCERN items, performing particularly poorly in the first section of the DISCERN instrument, which focused on assessing the reliability of the information presented on the website. Across most of the websites, the majority performed better on the second section of the DISCERN instrument, which assesses the quality of the information presented on the website, compared to the first section. All DISCERN scores for each of the eligible websites can be found in Table 2.

**Table 2:** DISCERN instrument ratings

Section			SECTION 1 Is the publication reliable?								SECTION 2 How good is the quality of information on treatment choices?							SECTION 3 Overall Rating of the Publication			HONcode Search
**DISCERN Question**		**Website Category: Health Portal (HP), Professional (Prof), Online TV (OTV), Government (Gov), Online Magazine (OM)**	**1. Are the aims clear?**	**2. Does it achieve its aims?**	**3. Is it relevant?**	**4. Is it clear what sources of information were used to compile the publication (other than the author or producer)?**	**5. Is it clear when the information used or reported in the publication was produced?**	**6. Is it balanced and unbiased?**	**7. Does it provide details of additional sources of support and information?**	**8. Does it refer to areas of uncertainty?**	**9. Does it describe how each treatment works?**	**10.Does it describe the benefits of each treatment?**	**11. Does it describe the risks of each treatment?**	**12. Does it describe what would happen if no treatment is used?**	**13. Does it describe how the treatment choices affect overall quality of life?**	**14. Is it clear that there may be more than one possible treatment choice?**	**15. Does it provide support for shared decision-making?**	**16. Based on the answers to all of the above questions, rate the overall quality of the publication as a source of information about treatment choices**	**Standard Deviation of Overall Score (Q16)**	**DISCERN Score (Sum of Q1-Q15)**	**Is it certified by HONcode (Yes/No)**
WebMD	https://www.webmd.com	HP	5.00	5.00	4.50	5.00	4.00	5.00	4.50	4.50	5.00	5.00	3.50	4.50	4.00	5.00	4.50	5.00	0.00	69.00	Yes
Diabetes Self Management	https://www.diabetesselfmanagement.com	HP	5.00	5.00	5.00	1.50	5.00	5.00	4.50	3.50	5.00	5.00	5.00	4.50	3.50	5.00	5.00	4.50	0.71	67.50	Yes
Diabetes.co.uk	https://www.diabetes.co.uk	HP	5.00	5.00	5.00	3.00	4.50	5.00	4.00	5.00	4.50	4.50	3.00	4.00	4.50	5.00	4.50	4.50	0.71	66.50	No
Very Well Health	https://www.verywellhealth.com	HP	4.50	5.00	4.50	5.00	5.00	5.00	3.50	3.50	5.00	4.50	3.00	4.50	2.50	5.00	4.50	4.00	0.00	65.00	Yes
University of Minnesota	https://www.takingcharge.csh.umn.edu	Prof	5.00	5.00	4.50	5.00	3.00	5.00	5.00	5.00	5.00	5.00	2.50	1.50	3.50	5.00	5.00	4.00	0.00	65.00	Yes
Diabetes in Control	http://www.diabetesincontrol.com	Prof	5.00	5.00	5.00	5.00	5.00	5.00	3.50	2.50	4.50	4.50	4.00	3.50	3.50	5.00	4.00	4.50	0.71	65.00	No
Medical News Today	https://www.medicalnewstoday.com	HP	5.00	5.00	5.00	5.00	5.00	4.50	4.50	3.50	5.00	5.00	3.50	1.50	2.50	5.00	4.50	4.00	0.00	64.50	Yes
EndocrineWeb	https://www.endocrineweb.com	Prof	5.00	5.00	5.00	5.00	5.00	5.00	3.50	3.50	4.00	5.00	2.50	2.50	3.00	5.00	4.50	4.50	0.71	63.50	Yes
Everyday Health	https://www.everydayhealth.com	HP	5.00	5.00	5.00	4.00	5.00	5.00	4.00	4.50	5.00	5.00	2.50	1.50	3.50	5.00	3.00	4.00	0.00	63.00	Yes
Healthline	https://www.healthline.com	HP	5.00	4.50	4.50	4.00	4.50	3.50	4.00	3.50	5.00	5.00	3.00	1.50	3.50	4.50	5.00	4.00	0.00	61.00	Yes
Totally Health	https://www.totallyhealth.com	HP	5.00	4.50	5.00	1.50	1.50	5.00	3.50	4.50	5.00	5.00	4.50	1.50	4.50	5.00	4.00	4.00	0.00	60.00	No
Diabetes Australia	https://www.diabetesaustralia.com.au	Prof	5.00	5.00	5.00	1.50	4.00	3.00	4.50	5.00	2.50	2.50	3.50	2.50	4.00	4.50	5.00	4.50	0.71	57.50	No
Endocrinology Network	https://www.endocrinologynetwork.com	Prof	2.50	2.50	4.50	5.00	5.00	5.00	4.00	5.00	4.50	5.00	3.50	1.50	2.50	5.00	1.50	4.00	0.00	57.00	No
Dr.Axe	https://draxe.com	Prof	5.00	5.00	4.50	3.00	3.50	2.50	1.50	2.50	5.00	5.00	4.50	3.50	4.50	5.00	1.50	4.00	0.00	56.50	No
Chelation	https://chelation.me	Other	1.00	N/A	4.50	5.00	5.00	4.50	1.50	4.50	5.00	5.00	4.50	3.50	3.50	5.00	4.00	4.00	0.00	56.50	No
University of Rochester Medical Centre	https://www.urmc.rochester.edu	Prof	3.50	4.50	4.00	1.50	2.00	5.00	4.00	5.00	5.00	5.00	3.50	2.50	1.50	4.50	4.50	4.00	0.00	56.00	No
Stamford Health	https://www.stamfordhealth.org	Prof	5.00	4.50	5.00	1.50	4.50	3.50	2.50	1.50	3.00	5.00	3.50	2.50	2.50	5.00	4.50	4.00	0.00	54.00	No
On Track Diabetes	https://www.ontrackdiabetes.com	HP	3.50	4.50	4.00	3.00	4.00	3.00	3.00	3.00	3.00	3.50	3.00	3.00	3.50	5.00	3.50	3.50	0.71	52.50	No
Conscious Lifestyle Magazine	https://www.consciouslifestylemag.com	OM	2.50	2.00	4.50	4.50	1.50	3.50	4.50	1.50	5.00	5.00	4.00	1.50	3.50	4.50	1.50	3.00	0.00	49.50	No
Today’s Dietitian	https://www.todaysdietitian.com	OM	2.50	4.50	4.50	5.00	4.50	4.50	1.50	3.50	4.00	2.50	2.50	3.00	2.50	2.00	1.00	3.00	0.00	48.00	No
Institute for Natural Medicine	https://naturemed.org	HP	5.00	4.00	4.50	5.00	2.50	3.50	4.50	1.00	1.50	1.50	1.00	3.00	1.50	2.50	4.00	3.00	0.00	45.00	No
Practo	https://www.practo.com	HP	3.50	3.50	4.00	1.50	2.5	2.50	3.50	1.50	2.50	4.50	1.50	1.00	2.50	4.50	2.50	3.00	0.00	41.50	No
Diabetic Nation	https://diabeticnation.com	HP	1.00	N/A	4.50	1.50	3.00	4.50	1.50	1.50	4.50	4.50	1.50	1.50	3.00	4.50	4.50	3.00	0.00	41.50	No
Alt-Med	http://www.alt-med.org	Prof	3.00	3.00	3.50	1.50	1.00	2.50	1.00	2.00	4.50	4.00	1.50	1.50	3.50	3.50	4.00	3.00	0.00	40.00	No
The Health Site	https://www.thehealthsite.com	HP	1.00	N/A	4.00	1.50	3.50	3.50	2.50	1.50	5.00	5.00	2.50	1.00	1.50	4.50	1.00	2.50	0.71	38.00	No
Association of Traditional Medicine and Acupuncture UK	https://www.atcm.co.uk	Prof	4.50	4.00	2.50	1.50	1.50	2.50	1.50	1.50	3.00	5.00	1.50	3.50	2.50	1.50	1.50	3.00	0.00	38.00	No
Dr. Kirsten Smith MD	https://www.drsmithnd.com	Prof	2.00	2.00	2.50	1.50	1.00	2.50	1.50	1.00	4.00	4.00	1.00	4.50	1.50	4.50	1.50	2.00	0.00	35.00	No
Onyx Integrative Medicine and Aesthetics	https://onyxintegrative.com	Prof	3.00	2.00	2.50	1.50	1.50	2.50	1.50	1.00	1.50	1.50	1.00	1.50	4.50	2.50	4.50	2.00	0.00	32.50	No
Canadian College of Naturopathic Medicine	https://rsnc.ca	Prof	1.00	N/A	3.50	1.50	1.50	2.50	3.00	1.00	3.00	3.50	1.00	1.00	2.00	4.00	3.50	2.00	0.00	32.00	No
NDTV Food	https://food.ndtv.com	OTV	2.00	2.50	2.50	1.50	2.50	3.50	1.50	1.00	1.05	3.50	1.50	1.50	2.00	3.00	1.00	2.00	0.00	31.00	No
**TOTAL Means**			3.70	4.13	4.25	3.08	3.40	3.92	3.12	2.93	4.03	4.30	2.78	2.48	3.03	4.33	3.45	3.55	0.17	52.40	
**TOTAL Standard Deviations**			1.50	1.09	0.82	1.61	1.45	1.03	1.26	1.50	1.20	1.06	1.19	1.19	0.95	1.01	1.43	0.86	0.30	12.10	

### Trends identified across resources assessed

#### Reliability issues

Question four of the DISCERN instrument assessed the reliability of the sources used to present information on the websites, particularly assessing whether the information provided by the website is supported by a source that readers can identify. Many eligible websites scored very poorly on this item. Of the 30 websites, 17 (56.7%) of them scored a 3 or lower.

#### Clear treatment options with benefits

Questions nine and 10 of the DISCERN instrument assessed whether the websites thoroughly discussed how the individual treatments work, and if the benefits of each of the treatments are stated, respectively. The majority of the websites scored highly on these two questions, with 21 out of 30 (70.0%) websites for question nine, and 23 out of 30 (76.7%) websites for question 10, scoring 4 or higher. Most of the websites presented many individual treatment options and listed the benefits of the treatments with explanations and evidence.

#### Multiple treatment options presented

Question 14 of the DISCERN instrument assessed whether the websites considered a variety of treatment options for T2DM. The majority of the websites scored highly on this question; out of the 30 websites, 24 (80%) scored a 4 or higher for this item. Beyond presenting CAM options for the treatment and/or management of T2DM, the websites emphasized the importance of there being multiple treatment choices to manage T2DM symptoms and elaborated on different therapy options.

#### Highly scoring websites for patients and consumers

For the purpose of this study, we defined highly scoring websites are those with an overall DISCERN score of 60 or higher and that have a score of at least four out of the possible five for the mean of the first 15 questions of the instrument. Out of the 30 eligible websites, only 11 met this criteria. Nine of the 11 websites scored a 4.5 or higher for question 5, which focused on assessing whether the websites information was unbiased and balanced. Additionally, all 11 websites scored a 4.5 or higher on question 10 of the instrument, which focused on identifying whether the website presented all treatment options and described the benefits of each treatment. The highly scoring websites performed well on this question because they presented treatment options clearly, used subheadings for each treatment, and provided details under each subheading outlining the intended benefits of each treatment. Each highly scoring website also scored a 4.5 or higher on question 14, which assessed whether more than one treatment choice was presented. Detailed characteristics of these highly scoring websites are shown in Table 3.

**Table 3:** Highly scoring websites for patients and consumers

Website Name	URL	Summed DISCERN Score (out of 75 total points)	Mean DISCERN Score for Q16	Website Category	Target Audience	Frequency of Updates
WebMD	https://www.webmd.com/diabetes/natural-remedies-type-2-diabetes	69.00	5.00	Health Portal	Patients/public	Update frequency not available
Diabetes Self Management	https://www.diabetesselfmanagement.com	67.50	4.50	Health Portal	Healthcare providers, patients/public	Updated occasionally
Diabetes.co.uk	https://www.diabetes.co.uk	66.50	4.50	Health Portal	Healthcare providers, patients/public	Updated occasionally
Very Well Health	https://www.verywellhealth.com	65.00	4.00	Health Portal	Healthcare providers, patients/public	Website is updated monthly
University of Minnesota	https://www.takingcharge.csh.umn.edu	69.00	4.00	Professional	Healthcare providers, researchers, patients/public	Update frequency not available
Diabetes in Control	http://www.diabetesincontrol.com	67.50	4.50	Professional	Researchers, healthcare professionals	Website is updated occasionally
Medical News Today	https://www.medicalnewstoday.com	66.50	4.00	Health Portal	Patients/public	Update frequency not available
Endocrineweb	https://www.endocrineweb.com	65.00	4.50	Professional	Healthcare providers, patients/public	Website is updated occasionally
Everyday Health	https://www.everydayhealth.com/	69.00	4.00	Health Portal	Patients/public	Update frequency not available
Healthline	https://www.healthline.com/	67.50	4.00	Health Portal	Healthcare providers, patients/public	Update frequency not available
Totally Health	https://www.totallyhealth.com	66.50	4.00	Health Portal	Healthcare providers, patients/public	Update frequency not available

## Discussion

The purpose of this study was to identify and assess the quality of websites providing CAM consumer health information for the treatment and/or management of T2DM. Many patients consult online resources with the intention of self-diagnosis and seeking treatment options for their health conditions [17], and patients with T2DM searching for information about CAM are no exception [7]. It is important for healthcare providers to be aware of the quality of consumer health information presented on these websites, as this can better allow them to engage in discussions with their patients regarding what constitutes a high-quality online resource and to facilitate shared decision-making.

Of the 480 websites that were identified, 30 met the eligibility criteria and were included in the present study. Across all eligible websites, the mean summed DISCERN score was 52.40 (SD = 12.11), and the mean overall quality score (question 16) was 3.55 (SD = 0.86). The total mean scores of questions 2, 3, 9, 10 and 14 across all assessed websites exceeded four, while all 30 websites scored a mean of below 4 for the remaining questions. Eleven out of the 30 websites scored an overall DISCERN score of 60 or higher. It was found that variability in the quality of information existed across the eligible websites, based on our DISCERN instrument appraisals.

The majority of the eligible websites scored well on the reliability section of the DISCERN instrument, however, it should be noted that one general trend included that low quality sources, as defined by the DISCERN instrument, were referenced by some websites. In contrast, many of the websites scored poorly in the quality of the information section of the DISCERN instrument. A common trend, especially across the websites categorized as professional or health portal, included the highlighting of the academic titles/degrees held by website authors, despite the provision of biased or unreliable information, which in combination could be misleading to patients. Websites providing high-quality information should instead be based upon evidence informed by the medical literature, such as clinical practice guidelines [18, 19]. 

Our findings can be compared to studies which assessed consumer health information on similar topics. Weitzman et al. examined the quality of information available on social networking websites regarding diabetes mellitus in 2010. They found that many of the websites they assessed contained misinformation about diabetes treatments, including the provision of unfounded ‘cures’; only 30% of the websites they assessed were considered to be of high quality with respect to the content and information presented [20]. They also reported that many of the online resources available for T2DM lacked disclaimers and failed to communicate to patients the importance of discussing treatment options with their physicians [20]. Another study conducted in 2019 by Kloosterboer et al. assessed the quality of information available online for patients with regards to diabetic retinopathy using *JAMA* benchmarks [21]. They concluded that the quality of information present online for diabetic retinopathy was both low and difficult for patients to interpret [21]. Another study conducted a content analysis of popular American diabetes websites and concluded that while they were engaging to readers, they often lacked credibility and in-depth information for caregivers and providers [22]. Conclusions derived from previous literature indicate that the quality of information found online surrounding the topic of T2DM and diabetes in general is low and mirrors our findings which found that the quality of CAM-specific T2DM online information is also low.

With respect to the total means of individual DISCERN question scores across all eligible websites, the lowest total mean scores were observed for questions 8, 11 and 12. These questions assessed the following characteristics respectively: the validity of evidence presented for therapies within the websites; whether the website authors included appropriate assessments of the risks of each therapy suggested within the website; and whether the website authors thoroughly present the implications for choosing not to use a treatment for the disease or condition. Based on our subset of websites assessed, some common indicators that a website contains low quality information included: the website presenting therapy options for a disease or condition without citing supporting scientific evidence, failing to present the health impacts or risks of using a specific therapy for a health condition, or failing to present the implications of choosing not to use treatments or therapies for a health condition.

The present study’s findings can also be compared to the literature written about the quality of CAM-related consumer health information. A study conducted by Chen et al. in 2018 identified that the quality of information presented on complementary and integrative health websites was variable; many websites scored well in measures of ownership and navigability, but scored poorly on measures related to source attribution and authorship, bringing into question the reliability of sources used [23]. In the study, Chen et al. concluded that the wide range of website types showed that the public experiences information through a variety of media and it would be helpful to investigate how the presentation of this content varies depending on the medium [23]. Bessel et al. used the DISCERN instrument to assess the quality of information presented online for non-prescription and complementary medicines [24]. They found that consumers that use online resources to collect information about non-prescription and complementary medicines had insufficient access to reliable information and were unable to make informed health decisions about the medications they sought [24]. Three additional studies assessed the quality of online consumer health information at the intersection of CAM and low back pain, arthritis, and neck pain, respectively using the DISCERN instrument, finding that the quality of information was variable across different websites [25–27].

Due to the high prevalence of CAM use in patients with T2DM [28], the discussion between practitioner and patient regarding the safe and effective use of such therapies holds great importance [29]. The vast amount of information present online may present a burden to patients seeking to make informed decisions about their own health [29]. A study found that patient perceptions of control over their health accounted for increased trust in the patient-physician relationship. Trust was higher when patients felt that they had control over their health and perceived communication was participative [30]. Another study found that a high proportion of T2DM patients failed to discuss their use of CAM with their physicians [31]. The present study’s findings highlight the importance for healthcare providers to be aware of the quality and reliability of information online regarding CAM treatment options for T2DM, as this can help guide their discussions with their patients. Healthcare providers should be aware of their patients' information-seeking behaviour, assist them in navigating through the content they encounter online, and provide them with resources containing trustworthy and reliable information. Furthermore, a need exists for researchers, healthcare providers and educators to develop guidelines that address these recent and emerging issues associated with online patient health information, raise awareness around online misinformation, and assist patients in identifying reliable online resources [32–35].

### Strengths and limitations

One strength of our study included the use of the DISCERN instrument, which has been found to be valid and reliable in assessing the quality of consumer health information. Additionally, we pilot tested both the data extraction and the application of the DISCERN instrument, which improved the standardization of reporting and inter-rater reliability. Another strength includes the fact that we searched four countries across Google, which allows our findings to be more applicable across different jurisdictions. A limitation of this study was the cross-sectional nature of the study; information online is subject to continual change, thus our study only captured the quality of information on this health topic at a snapshot in time. Lastly, because we also only included and assessed English-language websites, our study's findings may not necessarily be generalizable to the quality of information written in other languages online.

## Conclusion

The internet provides an avenue for the rapid dissemination of consumer health information. Patients with T2DM often search for such information online, including that which pertains to CAM and T2DM, and may use this information to guide the decisions they make about their personal health. The purpose of this study was to identify and assess the quality of information on websites providing CAM consumer health information for the treatment and/or management of T2DM. Following the quality assessment of eligible websites using the DISCERN instrument, it was found that, in general, our subset contained low quality consumer health information; most notably, they often failed to provide adequate references to support their health statements. Healthcare providers attending to patients with T2DM should be aware of the low quality of such websites in order to better discuss and promote shared decision-making with patients inquiring about or using CAM.

## Data Availability

All relevant data are included in this manuscript.

## Abbreviations

CAM: complementary and alternative medicine
T2DM: type 2 diabetes mellitus
